# Perception of Affordance during Short-Term Exposure to Weightlessness in Parabolic Flight

**DOI:** 10.1371/journal.pone.0153598

**Published:** 2016-04-20

**Authors:** Aurore Bourrelly, Joseph McIntyre, Cédric Morio, Pascal Despretz, Marion Luyat

**Affiliations:** 1 PSITEC—Psychologie: Interactions Temps Émotions, EA 4072, Univ. Lille, F-59000, Lille, France; 2 CNPP—Centre de Neurophysique, Physiologie et Pathologie, UMR 8119, CNRS, Univ. Paris Descartes, F-75270, Paris, France; 3 Tecnalia Health Research Laboratories, E-20009, San Sebastian, Spain; 4 IKERBASQUE Research Foundation, E-48013, Bilbao, Spain; 5 Decatlhon SportsLab, F-59650, Villeneuve d’Ascq, France; 6 INSERM CIC-IT 807, F-5900, Lille, France; University of Turin and the Italian Institute of Technology, ITALY

## Abstract

We investigated the role of the visual eye-height (VEH) in the perception of affordance during short-term exposure to weightlessness. Sixteen participants were tested during parabolic flight (0g) and on the ground (1g). Participants looked at a laptop showing a room in which a doorway-like aperture was presented. They were asked to adjust the opening of the virtual doorway until it was perceived to be just wide enough to pass through (i.e., the critical aperture). We manipulated VEH by raising the level of the floor in the visual room by 25 cm. The results showed effects of VEH and of gravity on the perceived critical aperture. When VEH was reduced (i.e., when the floor was raised), the critical aperture diminished, suggesting that widths relative to the body were perceived to be larger. The critical aperture was also lower in 0g, for a given VEH, suggesting that participants perceived apertures to be wider or themselves to be smaller in weightlessness, as compared to normal gravity. However, weightlessness also had an effect on the subjective level of the eyes projected into the visual scene. Thus, setting the critical aperture as a fixed percentage of the subjective visual eye-height remains a viable hypothesis to explain how human observers judge visual scenes in terms of potential for action or “affordances”.

## Introduction

Most of the time we guide our activities in an adaptive manner and without incident. During locomotion, for example, we select support surfaces that can carry our weight and accommodate our posture while at the same time we avoid obstacles. The “affordance” neologism proposed by J. J. Gibson [1] codifies this faculty of an animal to guide its behavior by hypothesizing that the animal perceives what the environment has to offer in terms of action possibilities. An affordance can be defined as the potentials for action for an animal with respect to the properties of an object, surface or event, combined with the properties of the animal itself in terms of physical characteristics (height, weight, energy potential) and action capabilities. Since Gibson's proposal, a growing body of research has studied the perception of affordances in humans in normal terrestrial conditions [2–8].

One pertinent example of affordances is embodied by a study by Warren [6] in which the authors demonstrated a perception of affordance by humans in the estimation of a stair’s “climbability”. Participants viewed stairs of different heights and judged which ones they could climb in a normal fashion. The results revealed that participants’ perceptual judgments were consistent with respect to their actual stair-climbing capabilities computed from a biomechanical model of climbability. Furthermore, the maximum riser height which was perceived to afford “climbing on” could be expressed as a constant proportion of leg length (π = Riser height / leg length = 0.88) whatever the actual height of the observer. Subsequently, Mark [2] demonstrated that judgments of stair climbability (and also of chair “seatbility”) were based on information that are available in the optic array and that are scaled to a reference visual angle specific to each subject, the visual eye-height (VEH) defined below.

**Fig 1:**
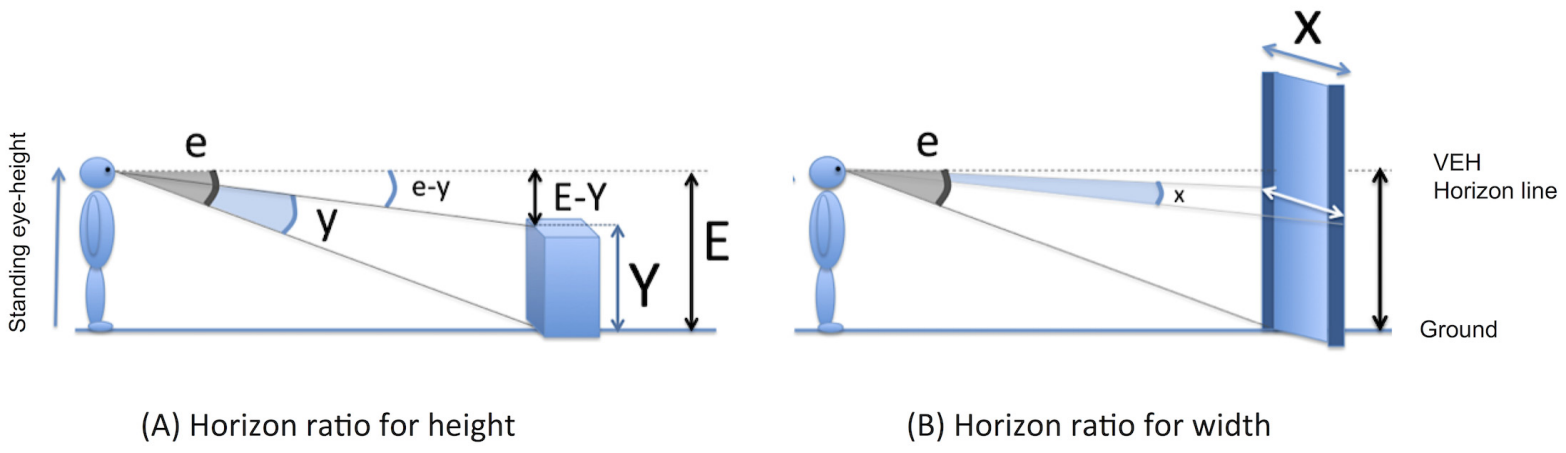
Illustration of the geometrical rules of optics that express the height and width of an object as a proportion of the VEH. A) Y is the object’s total height, E the distance between the bottom of the object and the horizon, and y and e are the respective heights expressed as visual angles. The height of the object (Y) can be expressed as a proportion of the observer’s VEH (E) by means of the *VEH ratio* defined as: Y/E = 1—tan(e-y) / tan(e) [5,11,13]. B) X is the object’s total width, E the distance between the bottom of the object and the horizon, and x and e are the same quantities expressed as visual angles. The width of the object (X) can be expressed as a proportion of the observer’s VEH (E), by means of a VEH ratio defined as: X/E = 2 tan(x/2) / tan(e) ≈ x / e [13].

Eye-height can be defined as the actual, standing (i.e. anthropometric) height of an observer’s line of gaze above the level of the floor. Standing eye-height can be perceived by vestibular and proprioceptive cues [9–11] but also by visual information. Indeed, in an open field environment, the convergence of ground texture and the convergence of lines that vanish at the horizon specify standing eye-height. This latter information, given by the optic array, defines what it is called the visual eye-height (VEH). On Earth, standing eye-height and VEH are usually redundant, as illustrated in Fig 1. The geometrical rules of optics can thus express the dimension of an object (height and width) as a proportion of the observer’s VEH [5,12–14]. Moreover, standing eye-height bears a lawful link to the different parts of the body in any individual, including, for instance, leg length. Using VEH to determine the ratio "riser height / eye height" (Fig 1A) therefore also gives information about the ratio "riser height / leg length", allowing one to judge if the riser is climbable or not [2,6]. Similarly, perception of the width of an aperture in relation to the eye-height of the observer (Fig 1B) allows one to judge if the aperture affords passage or not [7,13,15,16]. Perceiving the different dimensions of the environment in relation to the visual eye-height of the observer (and as a consequence to the standing eye-height) thus allows him or her to make body-scaled action judgments; i.e. to perceive affordances.

In line with Mark's research [2], several studies have manipulated VEH artificially in order to test the relevance of the eye-height concept in the perception of affordances on Earth (pass-through-ability [7,13,15,16]; pass-under-ability [17,18]; step-onto-ability [2,6]; step-across-ability [19]; reach-toward-ability [20,21]). For instance, Warren and Whang [7] tested the use of VEH on the visual guidance of walking through apertures using a false-floor paradigm. They dissociated the observer’s VEH and true standing eye-height by raising artificially the level of the visual floor with respect to the actual standing surface. In the third experiment from their 1987 study [7], these authors asked participants to judge, without actually performing the action, whether they could pass through doorways with different widths, and they looked for effects of raising the visual floor on the passability judgment. They found that the critical aperture, i.e. the minimal width at which participants judged they could pass through without turning or scrunching the shoulders, depended on VEH, while perceived distance (verbal distance estimation) did not. Thus, decreasing VEH made apertures appear more passable (see also Wraga [16]).

Based on the studies mentioned above, one can conclude, therefore, that observers rely on VEH information to perceive passability in normal terrestrial conditions; but what about other, more unusual, circumstances such as spaceflight? On Earth, VEH can be used as a reference to judge body-scaled actions (i.e. to perceive the affordance) because the eye-height and the object to be perceived (e.g. an aperture) share a common surface: the ground (see Fath & Fajen [13]). In weightlessness, however, the individual is free to float at any height without constant contact with the floor. When an observer is unrestrained in this environment, eye-height, and as a consequence VEH, moves vertically with respect to the floor and thus becomes irrelevant to the scaling of body-related actions. This could lead one to abandon VEH as a perceptual strategy in weightlessness.

One might also expect an effect of gravitational conditions on body-scaled judgments based on recent research on perception in weightlessness. Intuitively, one might imagine that the perception of one’s posture with respect to the environment would be perturbed in weightlessness due to the loss of tonic otolith information about the tilt of the head with respect to gravity. Various studies have shown that observers become more dependent on visual cues in the absence of gravity while the body axis takes on an increased importance in defining the perceived vertical [22–26]. Indeed, astronauts are unable to produce a consistent “upright” posture in 0g in the absence of visual cues [27]. Less obvious are the effects of gravity on visual perception, where the notion of direct vestibular influences on the perception of retinal signals is not intuitive. Nevertheless, experiments on orbit and during parabolic flight have demonstrated the influence of gravitational cues on visual percepts such as the interpretation of ambiguous figures [28,29], the perception of object orientation [30,31] and the identification of an axis of symmetry [32–35]. Other studies have shown an influence of weightlessness on the perception of visual cues linked to whole-body motions [36,37]. Of particular interest to the question of object affordances are studies showing the effects of weightlessness on the perceived shape and size of visual objects [29,30,38–41]. Among them, changes in apparent dimensions of objects were found to take place in weightlessness, leading to underestimation of objects’ size and distance [42–45]. Specifically, when participants had to adjust the size of a rectangular box displayed on a laptop so that it looked cubical (that is, with an equivalent size in width, height, and depth), they made its height shorter, its width wider and its depth longer than on Earth. In other words, the authors suggested that participants perceived a normal cube to be taller, thinner and shallower in weightlessness [42,43]. What is not known, however, is whether these modifications in the interpretation of visual cues in weightlessness have any influence on the perception of the environment in terms of its affordances for action.

The aim of this study, therefore, was to investigate the role of VEH information in the perception of affordances during acute exposure to weightlessness in parabolic flight. We hypothesized that VEH would lose its saliency in weightlessness because the ground no longer provides a common reference to scale body related actions. Thus, we predicted that we should observe an effect of VEH manipulation on the body-scaled critical aperture of a doorway during tests performed on the ground, but that this effect should disappear or be strongly diminished in trials performed during the weightless phase of parabolic flight.

## Method

### Parabolic Flight Experiment

We implemented a virtual-reality adaptation of Warren and Whang’s false-floor paradigm in order to test whether body-related actions continue to be scaled to VEH in the absence of gravity. The task required the participant, who was "immersed" in a virtual scene projected onto the screen of a laptop, to adjust the opening of a virtual doorway until it was perceived to be just wide enough to pass through without turning or scrunching the shoulders.

#### Apparatus

**Fig 2:**
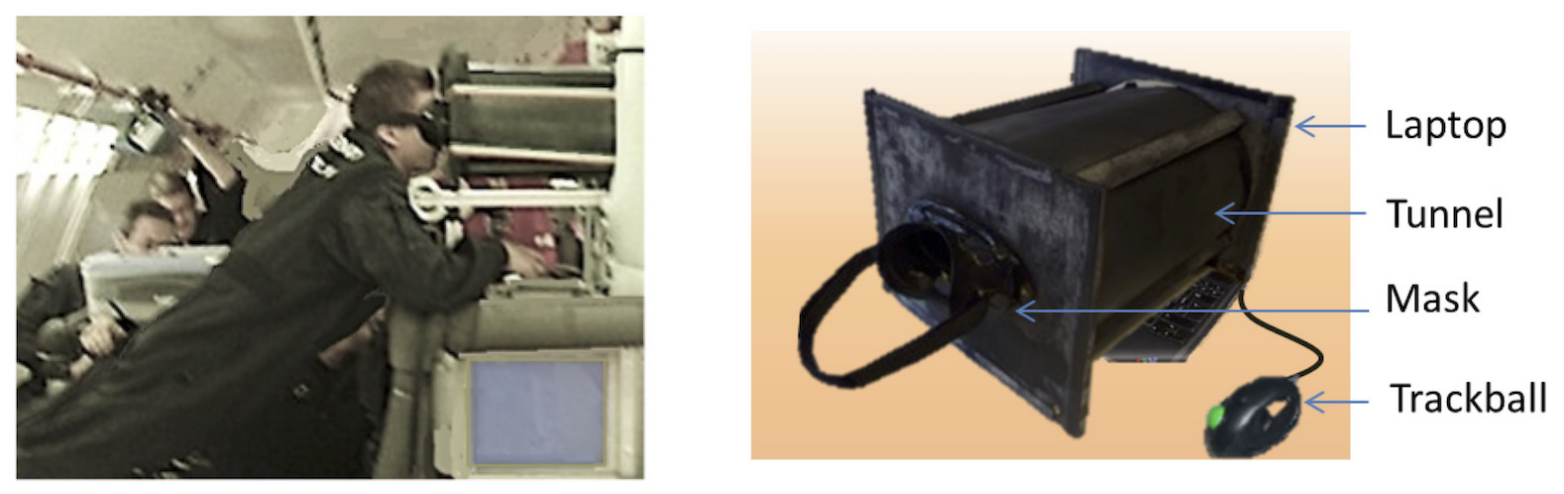
The measurement device. The measurement device consisted of a laptop mated with a tunnel and a diving mask. Participants looked at the visual scene displayed onto the screen. The visual scene was a picture of a room in which a doorway-like aperture was formed between two movable partitions. Perceptual adjustments were performed using a right-handed trackball.

The measurement device consisted of a laptop mated with a tunnel and a diving mask (Fig 2). The participants looked at images projected on the laptop screen (a 17-inch display) through the tunnel (26 cm height, 32 cm wide, 43 cm depth) in order to exclude other visual cues. The laptop was fixed to a rack attached to the wall.

#### The visual scene

**Fig 3:**
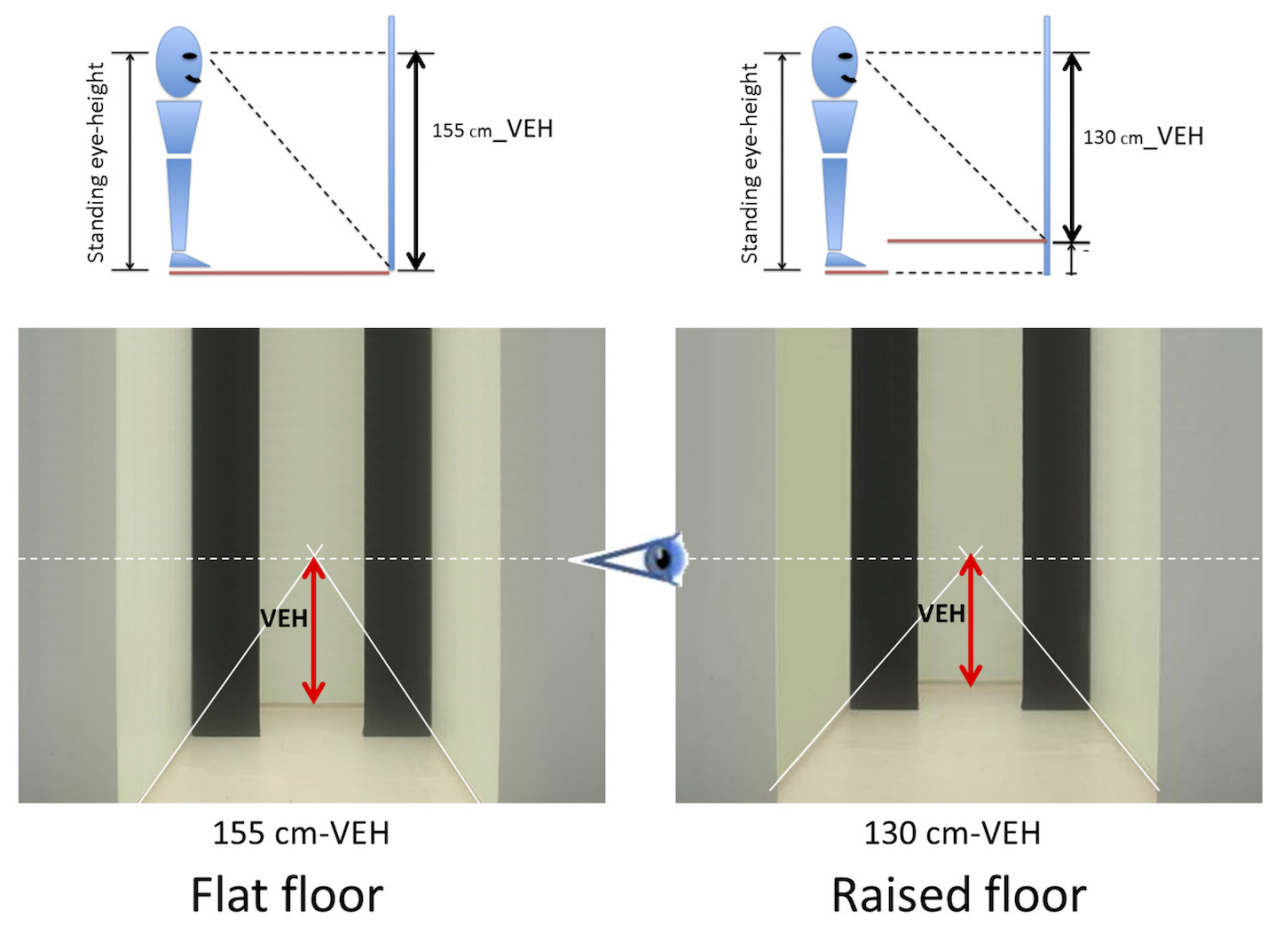
The visual scene. The visual scene was made from a photograph of a real room (220 cm horizontal X 260 cm vertical) in which a doorway-like aperture was formed between two movable panels. In the 130cm-VEH condition, the floor was raised about 25 cm. In this illustration, the superimposed solid white lines indicate the convergence lines from the lower middle of the photography toward the center of the screen. Note that the vanishing point remained at the center of the screen whatever the visual condition. The dotted line indicates the level of the eyes at the center of the screen. Neither the solid nor the dashed white lines were actually present in the visual scenes.

The visual scene was constructed from a photograph of a real room (220 cm horizontal X 260 cm vertical) in which a doorway-like aperture was formed between two black panels (see Fig 3). Photos of the aperture were taken at a distance of 7 m. Two snapshots of the scene were taken from different viewing heights in order to simulate a flat floor (155cm-VEH) and a raised floor (130cm-VEH).

**Fig 4:**
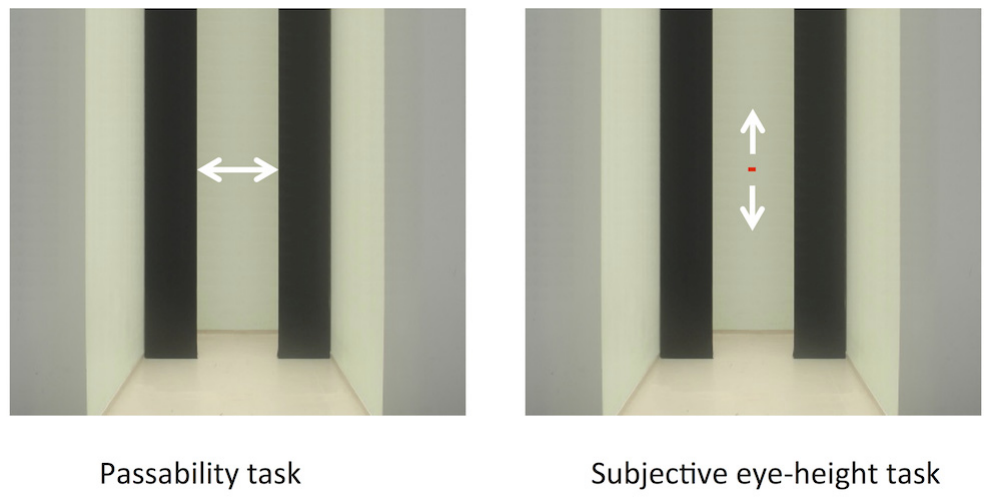
Passability and subjective eye-height tasks. In the passability task, participants were asked to adjust the opening of the doorway until it was perceived to be just wide enough to pass through without turning or scrunching the shoulders. The door could be adjusted by means of a hand-held trackball that controlled the lateral translation of the right and left black-painted movable panels to produce a doorway like aperture varying in width in an expanding or retracting manner. The measure obtained was the critical aperture (A, in cm) which is the minimum width of the aperture perceived by the subject to be “passable”. In the subjective eye-height task, participants adjusted a horizontal line to be at his or her perceived level of the eyes in the visual scene. The measure obtained was the so-called subjective eye-level, expressed as a deviation angle in degrees relative to the center of the screen, or reported as the subjective visual eye-height when computed in degrees relative to the visual floor.

Depth cues were available in these photos via the texture on the walls and floor and from the intrinsic vanishing point formed the convergence of the lines and edges of walls and floor from the lower middle of the photographs. The convergence lines from the ceiling were removed through digital modification of the images (Adobe Photoshop™). The photographs were then imported into the custom “VISION” software for building and running the experiment. The separation between the black panels was made to be adjustable in width by concomitant lateral translation of the panels through digital processing of the images. Thus, the participant was able to adjust the width of the aperture in an expanding or contracting manner (see Fig 4).

#### Procedure

Two tasks were performed, a passability task followed by a subjective eye-height task. Participants were initially familiarized with the tasks and the environment by performing 10 training trials. The tasks went as follows:

*Passability task (critical aperture)*: Participants were asked to adjust the opening of the doorway in the visual scene by manipulating a trackball until they perceived the aperture just wide enough to fit through without turning or scrunching the shoulders (critical aperture; see Fig 4). This required participants to imagine moving toward the door, at normal pace, considering their actual position and their environmental context. No further instructions were given concerning the mode of displacement to adopt (see Discussion). These adjustments were made in the two VEH conditions, 155cm-VEH and 130cm-VEH, described above. Six adjustments were done for each VEH condition, with the initial aperture set to 0 on three of the trials and to the maximum on the other three. The VEH conditions were presented in random order. It took around 2 s to respond to each stimulus and the following trial started the moment the participant had pressed the button to validate his/her previous trial.

*Subjective eye-height task*: Participants were asked to move a cursor (a red horizontal line subtending 0.08° of visual arc) up and down in the visual screen with a trackball until it reached the level at which he or she *perceived* his or her eyes in the visual scene (see Fig 4). These adjustments were made in three visual conditions presented in random order: neutral (blank mask), 130cm-VEH and 155cm-VEH. The 130cm-VEH and 155cm-VEH conditions were the same as those described for the passability task, with the aperture between the doors set at a fixed width of 90 cm. In the neutral condition, the participant observed a uniform white screen. Six adjustments were performed in each visual scene (3 from the lowest possible bar position and three from the highest). The mean of the 6 judgments within each visual condition was used for analysis.

#### Modulating gravity

The experiment was carried out during a campaign of parabolic flights sponsored by the Centre National des Etudes Spatiales (CNES) on board the Airbus A300 Zero-g. This aircraft is capable of flying parabolic trajectories during which the entire cabin is put in close-to-zero effective gravity (less than 10^−2^ g) for about 20 s. Subjects experienced ~20 s of hypergravity (1.6–1.8 g) just before and just after each weightless period as the aircraft pulled up to enter into the parabola and pulled out of the parabola and back into level flight, respectively. Each participant was tested twice: inflight during weightless phases of parabolic flight (0g) and inside the plane while on the ground in normal gravity (1g). The 1g condition was performed on the ground due to time constraints of the flights. Doing so also eliminated the potential influence of airplane vibrations and the unpredictable changes of gravito-inertial forces that would nevertheless occur during level, 1g flight [46].

At the beginning of each test, for both normal gravity and weightlessness, the participant stood in front of the laptop device with the head strapped to the diving mask. The participants were then required to hold onto the mask with the left hand and to use the right hand perform adjustments with a trackball. On the ground, subjects remained in this posture while performing the perceptual judgments. For the trials performed in parabolic flight, the participants were asked to keep the standing position with the feet on the floor during the hyper-g phases, firmly held and monitored by the experimenter. At the beginning of the weightless phases, participants assumed a quasi free-floating state by letting the legs float off of the floor so that their body reached an orientation, with the help of the experimenter, that varied from 45° to the almost horizontal, depending on the participant. Participants performed the perceptual judgments only during the 0g phase of the parabolas.

#### Participants

Sixteen volunteers (11 men and 5 women; mean age 33 ±8 years) participated in the parabolic flight experiment. Participants had vision that was normal or corrected-to-normal by lenses. Participants were not selected *a priori* regarding their stature. Their standing height ranged from 162 cm to 183 cm (mean = 174 cm; SD = 5.8). Their shoulder width ranged from 42 cm to 53 cm (mean = 46.5; SD = 2.99). They had no previous history of vestibular dysfunction or other neurological symptoms and were free from any known locomotor disorders (they all had had to pass a medical examination to be allowed to participate in parabolic flight). All were naïve with respect to the hypotheses of the experiment. The independent regional ethics committee (Comité de Protection des Personnes Nord Ouest III) approved the study and participants gave their written informed consent, in accordance with the Helsinki accords.

For 13 of the 16 participants this was their first exposure to parabolic flight; the others had experienced parabolic flight on different occasions. Due to motion sickness, 3 subjects were unable to perform the entire procedure. All 16 subjects performed the passability task in both 0g and 1g, while 13 out of 16 successfully completed the subjective eye-height task in both gravity conditions.

### Data analyses and statistics

*Measurements*: For the passability task, we computed the critical aperture (A), in centimeters, as the mean of the six judgments within each visual and gravitational condition. For the subjective eye-height task, the perceived eye-height was expressed as the visual angle measured in degrees relative to the center of the screen, hereafter called subjective eye-level. In the 130cm-VEH and 155cm-VEH conditions, the perceived eye-height was also reported in degrees relative to the visual floor, hereafter called subjective visual eye-height (sVEH). For each subject, his or her true standing height in the real world was also measured using a stadiometer while standing upright in normal gravity. Shoulder width, the widest frontal body dimension, was measured with an anthropometer from the tip of the left humerus (humeral greater tubercle) to the tip of the right humerus with the shoulders relaxed.

*Passability*: After having ensured that the application of ANOVAs was statistically appropriate (Shapiro Wilk's test assessed that the data were normally distributed), we performed a two-way ANOVA (2 gravity conditions * 2 VEH conditions) with repeated measures on the critical aperture. This analysis was based on data from all 16 subjects.

*Subjective eye-level and Subjective visual eye-height*: We compared the mean values of subjective eye-level for 0g and 1g in the neutral condition (blank mask) with Student’s pairwise t-test. For the subjective eye-levels reported in the presence of the visual doorway, we conducted a two-way ANOVA with repeated measures on the values obtained for 155cm-VEH and 130cm-VEH during both weightless and normal gravity sessions (2 gravity * 2 visual conditions), after having ensured that the application of ANOVA was statistically appropriate (Shapiro Wilk’s test). A similar two-way ANOVA was also applied to the subjective visual eye-height (sVEH) measured in degrees relative to the visual floor. The analyses of subjective eye-height task were conducted on the 13 participants who successfully completed this protocol in 0g.

*Reliance on VEH*: We determined the percent reliance of A on visual eye-height (%VEH) by comparing the *actual* and *predicted* diminution of the critical aperture when the visual floor was raised by 25 cm (i.e. between the 155 and 130 cm VEH conditions), computed as follows:

$$\%VEH=\frac{actual\;\Delta A}{predicted\;\Delta A}\times 100$$

$$actual\;\Delta A=A_{155cm}-A_{130cm}$$

ΔVEH=155−130

=25

$$predicted\;\Delta A=A_{155cm}\times \frac{\Delta VEH}{{VEH}_{155}}$$

$$=A_{155cm}\times \frac{25}{155}$$

A Student’s pairwise t-test was used to compare %VEH between the 0g and 1g conditions.

#### Correlation analyses

We performed Bravais-Pearson correlation analyses 1) between the participants’ shoulder width and critical aperture in the 155cm-VEH condition, to test whether the wider participants were those who showed the larger critical apertures; 2) between the participants’ true standing eye-height and the sVEH in the 155cm-VEH condition, to test whether the taller participants were those who reported the higher sVEH, and 3) between the adjustments of the critical aperture and the sVEH, to test whether the participants who produced the larger critical apertures were those who reported the higher sVEH. These analyses were performed on the 13 participants who successfully performed the two tasks (passability and subjective eye-height), for normal gravity and weightless session.

### Control Experiments

In our weightless experiments two main factors might have influenced the perception of the apertures, the lack of gravity *per se* and the semi-prone, floating posture that the subjects adopted in weightlessness. In order test how postural context may have contributed to the perception of passability in weightlessness, we performed additional experiments similar to the main protocol described above. In these two control experiments, performed only in normal gravity on the ground, subjects observed a virtual room on a video screen viewed through a mask and tunnel. The visual environment, constructed with 3D graphics software, consisted of a corridor with textured walls, ceiling and floor and with a doorway at the far end. The doorway was equipped with sliding panels that could be opened or closed to varying degrees by manipulating a trackball. The floor of the virtual room could be moved upward by 25 cm with respect to the walls and ceiling.

**Fig 5:**
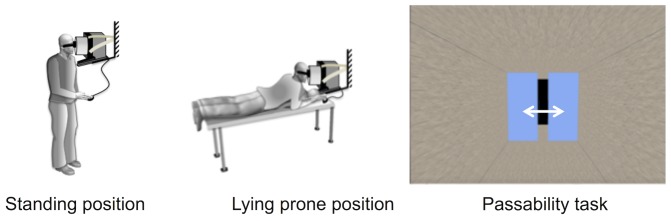
Subject posture and visual stimuli for the control experiments. In the first control experiment subjects performed the critical aperture and subjective eye-height tasks either in an upright standing posture or while lying prone on a cushioned table. In the second control experiment, performed only in the prone position, the viewpoint into the virtual room was either held static or displaced dynamically to evoke the visual sensation of floating freely within the 3D visual environment.

In the first control experiment we tested specifically for an influence of body posture on both the critical aperture and on the sVEH. Subjects performed the tests either in a normal, standing posture or while lying prone on a cushioned table, with head and shoulders propped up by leaning on their elbows (see Fig 5). In both situations the computer was maintained in a normal upright position and the axis of the tunnel (i.e. the viewing axis) was horizontal with respect to gravity. As in the main experiment, subjects were tasked with adjusting the sliding panels such that the aperture would allow them to pass without turning or scrunching their shoulders. Subjects performed 4 repetitions of the critical aperture adjustment for each level of the virtual floor and in each posture. The mean across these 4 repetitions was used for analysis. The two different postures (standing or prone) were tested in separate blocks, with the order counter-balanced across subjects. Presentation of the two different floor heights was randomized within each block. Subjects were also asked to perform the subjective visual eye-height task by adjusting a horizontal bar presented in the visual scenes. They performed 10 repetitions for each floor height in each postural condition. The critical aperture was subjected to a two way ANOVA with repeated measure (2 postures x 2 VEH conditions). A similar ANOVA was applied to the sVEH. Twenty subjects (10 males, 10 females) participated in this control experiment.

In the second control experiment we tested whether a floating “postural context” might influence the perceived critical aperture. In this experiments subjects were also asked to adjust the doorway to the critical aperture, as in the experiments described above. In contrast to the main experiment, however, subjects were instructed to imagine their movement toward the door while taking into account the current posture of their body. Given the prone posture, this implied a floating or swimming motion toward the doorway without contact of the feet on the ground. Going a step further in this regard, the viewpoint of the subject within the virtual room was either a) held static, corresponding to an observer who does not move within the room, or b) displaced dynamically, with smooth up/down and left/right movements of the viewpoint in the fronto-parallel plane. This latter condition was meant to evoke the visual sensation of floating freely within the 3D visual environment. The experiment was divided into two blocks, one for each postural context (static or dynamic) counterbalanced across participants. VEH varied randomly between the two possible values within each block. Sixteen adjustments were performed in each condition, leading to a total of 32 trials per block and 64 trials overall. The mean of the 16 judgments were subjected to a two way ANOVA with repeated measure (2 postures x 2 VEH conditions) on the critical aperture. Twenty subjects (10 males, 10 females) participated in this control experiment.

## Results

In debriefing, no participants explicitly reported noticing any changes between the 130cm-VEH and 155cm-VEH conditions. Overall, participants did not report any difficulties in performing the tasks. Globally, there was no great problem of dizziness or motion sickness during parabolic flight, except for three subjects who suffered of severe nausea during the subjective eye-height task (carried out towards the end of the flight) and who had to abandon the testing.

### Passability

**Fig 6:**
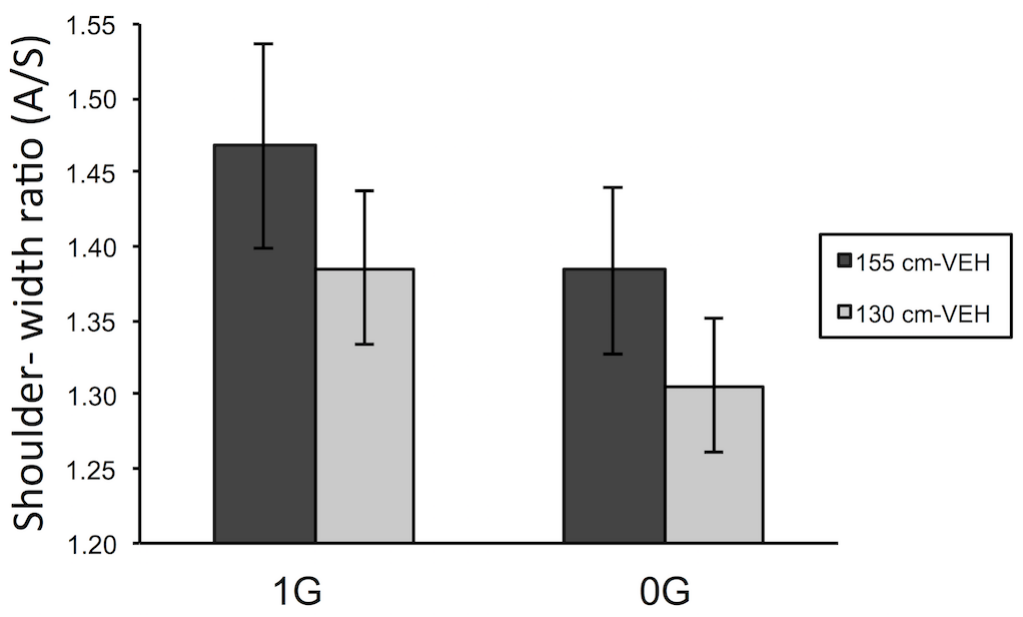
Shoulder-width ratio. Mean of shoulder-width ratios as a function of gravity session and visual condition (plotted with ± SEM; N = 16). SEM = Standard Error of the Mean, VEH = Visual Eye-Height. (see S1 File for raw data).

Mean values of critical apertures (A) and aperture-to-shoulder width ratios (A/S) are given in Table 1. The analysis showed a main effect of VEH on the critical aperture: F(1,15) = 14.26; p< 0.01; η^2^_p_ = 0.49; (1 − β) = 0.94. Specifically, for both weightless and normal gravity sessions, the mean of critical apertures was significantly lower for 130cm-VEH compared to 155cm-VEH (62.8 vs. 66.5, respectively). The analysis also showed a significant effect of gravity on the critical aperture: F(1,15) = 5.89; p< 0.05; η^2^_p_ = 0.28; (1 − β) = 0.62. For both the 130cm-VEH and 155cm-VEH conditions, the mean of critical apertures was significantly lower for trials performed in weightlessness compared to the normal gravity session (66.5 vs. 62.8, respectively). The interaction between the two factors was not significant: F(1,15) = 0.02; p = 0.89, meaning that we have no statistical evidence that the significant effect of VEH was different in 0g versus 1g. An ANOVA carried out on the shoulder-width ratios showed the same statistical conclusions, i.e. a significant effect of VEH and gravity and no cross effect (see Fig 6).

**Table 1:** Critical aperture and shoulder width ratio.

	Critical aperture (A)				Shoulder width ratio (A/S)			
Condition	1g		0g		1g		0g	
	mean	SD	mean	SD	mean	SD	mean	SD
155-VEH	68.4	12.0	64.5	10.2	1.47	0.23	1.38	0.18
130-VEH	64.6	14.2	61.0	11.9	1.39	0.28	1.31	0.21

Mean and Standard Deviation (SD) of the critical aperture (cm), the shoulder-width ratio for participants in the 155cm-VEH and 130cm-VEH conditions in normal gravity (1g) and weightlessness (0g). The shoulder-width ratios are given to allow direct comparison with the results of Warren and Wang [7]. N = 16 subjects. VEH = Visual eye-height, A = Critical aperture, S = Shoulder width.

### Subjective eye-level and Subjective visual eye-height

Data processing was carried out on the subjective eye-level, i.e. the mean deviation (in degrees) of the final position of the indicator bar relative to the center of the screen, for each of the three visual conditions (neutral, 155-VEH and 130-VEH) and on the subjective visual eye-height above the floor (sVEH) in the visual scene for the two conditions where a floor was present (155-VEH and 130-VEH). The results are summarized in Table 2.

**Table 2:** Subjective eye-level and subjective visual eye-height (sVEH).

	Subjective eye-level relative to the center of the screen (°)				Subjective visual eye-height (sVEH) relative to the visual floor (°)			
Condition	1g		0g		1g		0g	
	*mean*	*SD*	*mean*	*SD*	*mean*	*SD*	*mean*	*SD*
Neutral	0.03	2.99	-0.37	2.34	_	_	_	_
155-VEH	2.65	3.66	1.52	3.86	15.14	3.66	14.01	3.86
130-VEH	3.67	3.76	2.33	3.57	14.20	3.76	12.86	3.57

Mean and Standard Deviations (SD) of the subjective eye-level given in degrees relative to the center of the screen in the neutral, 155cm-VEH and 130cm-VEH conditions, in normal gravity (1g) and weightless (0g). The subjective visual eye-height is also reported in degrees relative to the visual floor (sVEH), when present. N = 13 subjects. VEH = Visual eye-height, sVEH = subjective visual eye-height.

Student’s t-test applied in the neutral condition (white mask) did not reveal a significant effect of gravity on the subjective eye-level: *t*(12) = 0.65; p = 0.53. Indications of subjective eye-level were about 0.03° on average in 1g and -0.37° on average in 0g, where positive or negative values indicate subjective levels above or below the center of the screen, respectively. In the trials where a visual floor and doorway were presented, ANOVA showed a significant effect of VEH on subjective eye-level for both weightless and normal gravity trials: F(1,12) = 21.3; p< 0.001; η^2^_p_ = 0.64; (1 − β) = 0.99. Specifically, subjective eye-level above the center was significantly higher for 130cm-VEH relative to 155cm-VEH, whatever the gravity condition (3.00° vs. 2.1°, respectively). The ANOVA also showed a significant effect of gravity on the subjective eye-level in these cases: F(1,12) = 8.8; p< 0.01; η^2^_p_ = 0.42; (1 − β) = 0.78, which was lower for weightless trials compared to trials performed in normal gravity (1g = 3.2° vs. 0g = 1.9°). The interaction between the two factors was not significant: F(1,12) = 1.0; p = 0.33.

**Fig 7:**
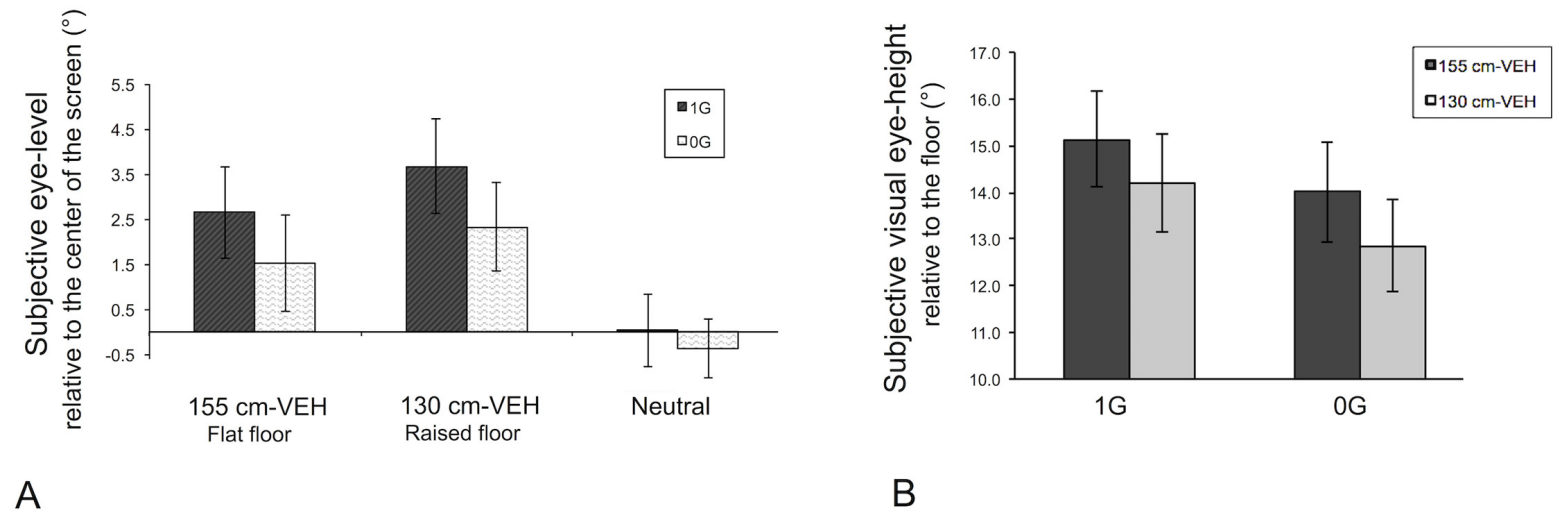
Subjective eye-level and subjective visual eye-height. A) Mean of subjective eye-level computed in degrees relative to the center of the screen as function of visual condition and gravity session (plotted with ± SEM; N = 13). B) Mean of subjective visual eye-height computed in degrees relative to the floor as function of gravity session and visual condition (plotted with ± SEM; N = 13). SEM = Standard Error of the Mean, VEH = Visual eye-height, sVEH = Subjective visual eye-height relative to the floor and in degrees. (see S1 File for raw data).

An ANOVA carried out on the subjective visual eye-height above the floor (sVEH) showed a significant effect of raising the floor for both weightless and normal gravity trials: F(1,12) = 27.7; p< 0.001; η^2^_p_ = 0.70; (1 − β) = 1. Specifically, sVEH was significantly lower for 130cm-VEH compared to 155cm-VEH for both gravity conditions combined (13.5° vs. 14.6°, respectively). The ANOVA also showed a significant effect of gravity on the sVEH: F(1,12) = 8.8; p< 0.01; η^2^_p_ = 0.42; (1 − β) = 0.78 such that sVEH was significantly lower for weightless trials (1g = 14.7° vs. 0g = 13.4°). The interaction between the two factors was not significant: F(1,12) = 1.0; p = 0.33 (see Fig 7).

It is perhaps worth emphasizing here for clarity the opposing effects of changing the visual floor height on the subjective eye-level with respect to the center of the screen and the subjective eye-height with respect to the visual floor. Even though raising the floor had the effect of raising the subjective level of the eyes with respect to the screen (i.e. with respect to the horizontal), this effect was not enough to counteract the reduction in the visual angle between the floor/door intersection and the center of the screen. Thus, raising the visual floor (VEH) still caused a reduction in the reported perceived visual eye-height (sVEH), but the reduction in sVEH was less than the expected value of 25 cm.

### A/VEH Scaling

The results of the analyses of the percent reliance on VEH are summarized in Table 3. The 25 cm change in VEH from 155 cm to 130 cm represents a relative change of 16.13% of the purported metric. If subjects fully scaled the critical aperture to VEH, one would expect the critical aperture (A) to change by a similar factor between the 155cm-VEH and 130cm-VEH conditions. For an average critical aperture of 68.4 cm for 155cm-VEH (see Table 1), the expected change in A would therefore be 11 cm. However, the actual diminution of the critical aperture in the raised floor condition was only about 3.8 cm (i.e. 68.4 cm—64.6 cm) indicating a reliance on VEH of about 34.4% for the normal gravity session. The expected change in critical aperture due to the raised floor was slightly different in weightlessness, because the critical aperture in the 155cm-VEH condition was slightly smaller in 0g compared to 1g. Raising the floor about 25 cm in the inflight session was therefore expected to reduce the critical aperture by 10.4 cm if VEH information alone was used. The actual diminution of the critical aperture in the raised floor condition was about 3.5 cm (i.e. 64.5 cm—60.0 cm), giving a relative reliance on VEH of about 33.8% for the inflight session. Student’s t-test showed no significant difference in these values between gravity conditions: *t*(15) = 0.15; p = 0.88. A corollary of this analysis is that the ratio of A to VEH did not remain constant across conditions.

**Table 3:** Predicted and actual diminution of the critical aperture.

	1g		0g	
	mean	SD	mean	SD
Predicted diminution (cm)	11.0	1.9	10.4	1.7
Actual diminution (cm)	3.8	4.9	3.5	4.8
Reliance on VEH (%)	34.4%		33.8%	

Means and Standard Deviations (SD) of the predicted and actual diminution of the critical aperture (cm) when raising the floor by 25 cm (i.e. the difference between 155cm-VEH and 130cm-VEH condition) in normal gravity (1g) and inflight session (0g). The reliance on VEH to scale the critical aperture is given in terms of percentage of expected VEH scaling. N = 16.

**Fig 8:**
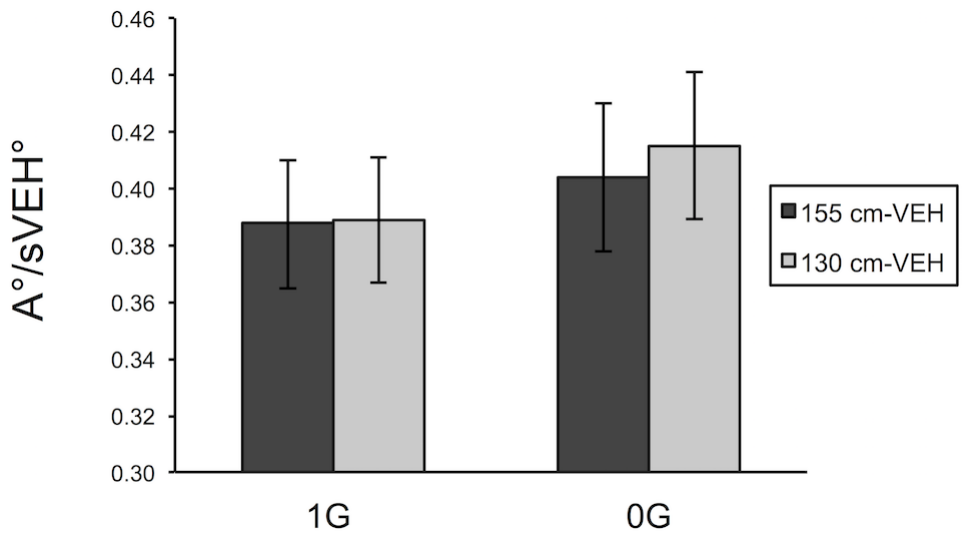
Ratio of A versus sVEH for each condition. Mean of A°/sVEH° ratio as function of gravity session and visual condition (plotted with ± SEM; N = 13). SEM = Standard Error of the Mean, A° = Critical aperture and sVEH° = Subjective visual eye-height relative to the floor, each expressed as a visual angle in degrees. (see S1 File for raw data).

We note, however, that changes in the sVEH between the 155cm-VEH and the 130cm-VEH conditions differed from the actual change of eye-height evoked by the changing floor level in the visual scene. Indeed, the critical aperture (A) and the sVEH, showed similar patterns of variation as a function of VEH (155 or 130) and gravity conditions (0g an 1g), as illustrated in Figs 6 and 7B. We therefore hypothesized that a perceptual strategy in which the critical aperture is scaled to the sVEH could explain the subjects’ perceptual responses across our experimental conditions. Indeed, the ratio of the critical aperture expressed as a visual angle (A°) to the subjective visual eye-height, also expressed in degrees (sVEH°), shown in Fig 8 appears to be much more constant across conditions. An ANOVA on the A°/sVEH° ratio showed no main effect of the VEH or gravity factors and no cross effect, suggesting that subjects may maintain a constant A°/sVEH° to choose the critical aperture (see Discussion).

### Correlation analysis

Correlation analyses applied to the results from the 155cm-VEH condition showed a significant positive correlation between the participants’ shoulders-width and the critical aperture (1g: r = 0.54, p< 0.05; 0g: r = 0.65, p< 0.05). Positive correlations were also found between participants’ true standing eye-height and subjective visual eye-height, reaching significance for the normal gravity session only (1g: r = 0.64, p< 0.05; 0g: r = 0.50, p = 0.09). The analysis also showed a significant correlation between the adjustments of the critical aperture and the adjustments of the subjective visual eye-height whatever the gravity session (1g: r = 0.66, p< 0.05; 0g: r = 0.57, p< 0.05).

### Control experiments

Mean of critical apertures (A) and subjective visual eye-height (sVEH) for the control experiments are given in Table 4. The analysis conducted on the first control experiment showed a main effect of VEH on the critical aperture: F(1,19) = 23.89; p< 0.001; η^2^_p_ = 0.56; (1 − β) = 1. Specifically, for both standing and prone conditions, the mean of critical apertures was significantly lower for 130cm-VEH compared to 155cm-VEH (63.7 cm vs. 68.7 cm, respectively). There was no significant effect of posture on the critical aperture: F(1,19) = 0.41; p = 0.53 and there was no interaction between the two factors: F(1,19) = 2.03; p = 0.17. The analysis performed on the sVEH showed a main effect of VEH: F(1,19) = 248; p< 0.001; η^2^_p_ = 0.93; (1 − β) = 1. Specifically, for both standing and prone conditions, the mean of sVEH was significantly lower for 130cm-VEH compared to 155cm-VEH (95.9 cm vs. 114.1 cm, respectively), as would be expected due to the raising of the visual floor. There was no significant effect of posture on the sVEH: F(1,19) = 3.55; p = 0.08 and the interaction between the two factors was not significant: F(1,19) = 0.02; p = 0.89.

**Table 4:** Critical aperture and subjective visual eye-height of the control experiments.

	Control Experiment #1								Control Experiment #2			
	Critical aperture (A)				Subjective visual eye-height (sVEH)				Critical aperture (A)			
	STANDING		PRONE		STANDING		PRONE		STATIC		DYNAMIC	
Condition	mean	SD	mean	SD	mean	SD	mean	SD	mean	SD	mean	SD
155-VEH	68.3	18.2	69.0	16.2	117.4	11.9	110.8	17.8	58.7	12.5	60.6	16.9
130-VEH	62.6	16.9	64.8	16.3	99.0	11.1	92.6	16.0	55.5	12.6	57.7	16.7

Mean and Standard Deviation (SD) of the critical aperture (cm) and subjective visual eye-height (cm) for participants in the 155cm-VEH and 130cm-VEH conditions in the control experiments. N = 20 subjects. VEH = Visual eye-height, A = Critical aperture, sVEH = Subjective visual eye-height.

The analysis conducted on the second control experiment showed a main effect of VEH: F(1,19) = 124.8; p< 0.001; η^2^_p_ = 0.87; (1 − β) = 1. Specifically, for both static and dynamic sessions, the mean of critical apertures was significantly lower for 130cm-VEH compared to 155cm-VEH (57 cm vs. 60 cm, respectively). There was no significant effect of mode of presentation (static or dynamic) on the critical apertures: F(1,19) = 1.51; p = 0.23. The interaction between the two factors was not significant: F(1,19) = 0.52; p = 0.46.

## Discussion

The main purpose of this study was to investigate the influence of gravity on perception of affordances for passing through apertures. Specifically, the question was to determine whether the perception of the threshold aperture for passability through a doorway remains scaled to VEH when the observer and the doorway no longer share the ground as a common and stable reference.

### Effects of VEH

In our experiments we found a significant effect of VEH on the mean critical aperture, whether expressed in absolute measure (cm) or as the ratio of the aperture opening to shoulder-width. We found that decreasing VEH in the visual scene by raising the visual floor made apertures of a given size appear more passable. Indeed, the mean of shoulder-width ratios was significantly lower for the 130cm-VEH condition compared to the 155cm-VEH condition. An aperture that appeared too narrow in the 155cm-VEH condition could appear to be passable when the VEH was reduced (130cm-VEH condition). Hence, everything happened as if the aperture appeared wider or the participant perceived himself to be thinner in the scene when VEH was smaller what is strongly supported by the reduced subjective eye-height in this condition. This result is consistent with the literature that found a global lowering of critical aperture with decreasing VEH [7,16]. Furthermore, the lack of any interaction effect between the VEH and gravity factors indicates that the effect of changing visual eye-height on the perceived critical aperture was equally strong in 0g and in 1g. Thus, our main hypothesis of a reduced or lack VEH effect in 0G is not confirmed by the data. Stable contact with a support surface (i.e. the floor) that is presumed to be the same as the visual floor is not an *a priori* condition for the application of a VEH strategy for the estimation of passability.

The other well-established hypothesis that VEH serves as a reference measurement explicitly states that the critical aperture for passage would be scaled in a fixed ratio to VEH, in line with Gibson’s principles of “direct perception”. Indeed, in the original study conducted by Warren and Wang, the authors found no evidence to the contrary, i.e. the ratio of critical aperture to VEH, each expressed in degrees, remained constant across the two different visual floor heights. In the study reported here we did not find such invariance. The reduction in critical aperture induced by a given rise in the visual floor was smaller than expected, as indicated by the low “relative reliance” on VEH. But the efficacy of a strategy to estimate the critical aperture in a real-world situation by keeping the A/VEH ratio constant is based on two assumptions, as illustrated in Fig 1. First, the observer must assume that the floor in the visual scene is the same floor as the one on which he or she is standing. We intentionally violated this condition by changing the floor height in the visual scene. The second assumption, however, is that the observer will project their eye-height into the visual scene parallel to the visual floor. Only if this second condition is met will holding A at a fixed proportion to VEH cause A to be properly scaled to shoulder width. In our experiments, however, changing both VEH and gravity had an effect on the subjective visual eye-height with respect to the floor, suggesting that the second of the two conditions has been violated (the eye-height is not projected parallel to the floor). The changes in subjective visual eye-height could in fact explain the unexpectedly small effect of changing floor height and the unexpected effect of gravity on the perceived critical apertures. Indeed, the lack of significant effects of either VEH or gravity on the A°/sVEH° ratio allows us propose a modification to the Warren and Whang’s hypothesis, i.e. that human observers judge critical apertures as a constant proportion of their *subjective* visual eye-height. This result led to the primary conclusion of our study, i.e. that the use of VEH (or more precisely sVEH) is a persistent strategy for the perception of affordances based on visual information, regardless of the postural conditions with respect to a stable support surface (1G versus 0G).

### Effects of Gravity

In addition to the effects of VEH on A that we observed, we also found that critical apertures were reduced by 3.75 cm on average in 0g. Compared to normal gravity, apertures appeared wider relative to the body when tests were performed in weightlessness. This observation is in line with the misperception of object dimensions in weightlessness found by Clément et al. [42,43], who showed that participants perceived a visually presented box to be taller, thinner and shallower in weightlessness compared to normal gravity. They, too, linked their results to a lower subjective eye-level [42,43] in weightlessness and suggested that this phenomenon might be due to a diminution of the pitch rotation of the eye due to changes in otolith organs stimulation in weightlessness [47,48]. The fact that weightlessness had no effect on subjective eye-level in our neutral condition (blank mask) seems to challenge this purely otolithic explanation. Nor could the semi-prone posture that subjects adopted in weightlessness provide the entire explanation because in our control experiment, standing or lying prone had no consistent effect on either A or sVEH. Rather, it would appear that the perception of spatial dimensions in the visual scene depends on an interaction between visual, proprioceptive and graviceptor cues.

One might ask, therefore, whether observers perceive apertures to be bigger or their own bodies to be smaller in weightlessness. From a strict interpretation of the term “direct perception” [1,49], this question perhaps has little meaning. Observers are purported to be perceive the scene directly in terms of potential for action, not explicitly in terms of shape or size. Nevertheless, one might ask whether there is a concomitant change in one’s conscious perception of one’s own size with respect to the world. As Gibson stated, “the information to specify the utilities of the environment is accompanied by information to specify the observer himself, his body, legs, hands, and mouth”. This reemphasizes that “to perceive the world is to co-perceive oneself” [1]. The measurements of sVEH suggest that subjects may indeed perceive themselves to be smaller in weightlessness. The proposed rationale is that raising the floor creates a new optical configuration wherein the proportion of the visual floor is more important in the scene compared to the flat floor (rules of optics). In the field of direct perception, this cue is characteristic of an observer’s point of view that would be smaller in the scene (e.g. from a smaller observer). This speculative idea is corroborated by (i) the smaller sVEH adjustments relative to the floor found when the floor is raised and (ii) the positive correlation between the adjustments of the critical aperture and the adjustments of sVEH. It is possible that the feeling of being smaller in the scene is also associated with a feeling to be thinner as if the body proportions are conserved. As a consequence, the critical aperture would be adjusted smaller in the raised-floor condition because the observer felt smaller and thinner.

Internal representations of the body based on somatosensory cues (i.e. the so-called “body scheme” [50]), are known to be modified in weightlessness. For instance, Clément et al. [51] showed large errors in determining the subjective egocentric vertical (an imaginary straight body midline running from the head to toes [52]) in the roll and pitch plane during acute exposure to weightlessness. And it is otherwise largely assumed that the internal representation of egocentric vertical has a prominent role in spatial orientation [53] and egocentric coding of objects while in weightlessness [54,55]. Less is known about the perception of body size in weightlessness. But the correlation analyses from our own study reported here suggest that participants kept a good sense of their relative body proportions in all conditions: the wider participants were those who produced the larger critical apertures, and those who produced the larger critical apertures were also those who reported the higher subjective visual eye-heights, in both normal and weightless conditions. Nevertheless, unlike in normal gravity, the taller participants were not systematically those who adjusted the higher subjective visual eye-height in weightlessness. This underlines the difficulties of perceiving one’s own viewing height when floating above the floor. It is conceivable, therefore, that perceived body size and, as a consequence, perception of environmental dimensions, could be modified in 0g. Another possible explanation is that the door are perceived to be more distant when VEH is reduced [56,57], although studies by Warren and Wang [7] suggest that distance and passability estimates are independent. Nevertheless, scaling of perceived critical aperture to sVEH (i.e. A°/sVEH° ratio) appears to be a persistent strategy for body-scaled actions, even in 0g.

### Confounding Factors

For practical reasons, we conducted our experiment using a form of virtual reality (subjects observed images on a screen rather than real objects), rather than asking subjects to observe real doorways. We found that the mean of shoulder-width ratios was greater than the physically minimal width required to adequately perform the task. Even on Earth, participants estimated to be just passable apertures that were actually 1.47 larger than their shoulder width, resulting in a non-negligible safety margin that was greater than those found previously in the literature (Warren & Whang [7] obtained a shoulder-width ratio of 1.16 using a forced-choice method). Although this cautious behavior may be exacerbated due to the nature of our visual scene, (a virtual scene based on a real picture), recent studies have shown that the perception of affordances, such as passing through apertures in virtual environments, is comparable to similar studies conducted in real world [58–60].

The analysis of percent reliance on the actual VEH to scale the perceived critical aperture showed similar values (about 34%) in both gravity conditions. This was lower than values found in the literature for a real room (about 65%[7] and 49% [16]) and for immersive head-mounted displays (about 77% [61]), but higher than that observed in a non-immersive display (TV/desktop screen set at 1 m from the observer), where no VEH effect was found at all [61]. This global lowering of percent reliance could be related to the artificial test situation in our study (looking through a mask without any visual cues from the environment, which is seldom the case in real life), but which does not necessarily mean that the virtual visual environment was sufficiently realistic [5,61]. Participants acted as if immersed in the virtual environment, rather than simply observing images on a computer screen. We note, as an aside, the importance of controlling for effects of experiment conditions on sVEH when interpreting experiments of this type. Wraga [5], for example, found a percent reliance on real VEH of about 44%, but also a less-than-expected (66%) reduction in sVEH due to the raised floor. Although Wraga found no statistical correlation between shifts in the perceived size of objects and shifts in sVEH, we did observe such a correlation, suggesting that passability judgments were based on the ratio of aperture visual angle to sVEH, rather than real visual eye-height, remains viable and should be further explored. On the other hand, the fact that the ratio of VEH scaling was never equal to unity indicates that subjects also relied on other sources of size information to make their judgments. The relative amount of ground texture covered by body segments was found to be one of these other cues [6].

We used parabolic flight as the means to test for an effect of gravity on the use of VEH in the judgment of passability through a doorway. One must therefore consider whether other characteristics of parabolic flight, such as the stress induced by this novel environment, the passages from 1g to 2g to 0g and back, or the administration of anti-nausea medications might explain the results. The fact that the critical aperture continued to be highly correlated with VEH in weightlessness, however, renders these questions moot. Had we observed a disappearance of the VEH effect in 0g, one might ascribe this hypothetical result to one of these ancillary factors. But because the VEH effect persisted in weightlessness we can confidently conclude that neither gravity nor a stable base of support are critical requirements for a VEH strategy to be employed.

The above-mentioned ancillary factors of parabolic flight could, however, be evoked to try and explain the overall reduction in critical aperture and the concomitant reduction in sVEH that we observed in 0g. For instance, in the weightless conditions subjects where in a floating, almost prone posture with respect to the video screen which might have changed the perception of either sVEH or A. But in our control experiments carried out in normal gravity we found no effect of posture (standing or lying prone) either on the overall critical aperture A or on the variations of A due to changes in VEH. These findings are in accordance to Wraga’s study [16] which showed no significant difference in judging the size of objects from standing and lying prone posture as long as the visual scene was observed from a similar viewpoint, as was the case in our study.

Administration of antinaupathique drugs commonly used to combat motion sickness in parabolic flight could conceivably have affected the orientation of the eyes in the orbit, through its actions on the inner ear, and thus the perception of sVEH within the visual scene. Although we did not explicitly control for such effects, other studies performed under the influence of antinaupathique drugs showed no effects of these medications on eye position [62] (reflecting a normal, physiological change in otolithic inputs brought about by the head orientation), visual side effects (visual acuity, and eye accommodation) [63], cognitive performance [64], and even the state of mood of the participants [65]. Furthermore, from our own study, weightlessness had no effect on sVEH measured in the neutral visual condition, even though subjects were under the influence of the same medications, if any, during these tests as well.

### Why use VEH in weightlessness?

One possible explanation for the persistence of VEH scaling in weightlessness is that the visual scene, which presented a well-defined floor, led the participants to imagine acting in a more usual ground context because it suggested a natural bipedal locomotion toward the door. Furthermore, it could be possible that the orientation of the visual scene, (which was perpendicular to the participant’s cephalo-caudal axis), would also lead the participants to judge actions in a terrestrial context. Regarding these previous concerns, one can legitimately ask whether the postural context may have contributed to the perception of passability in weightlessness. Our second control experiment addressed this issue. Here, subjects performed the passability task only in a prone position and the viewpoint into the visual scene was constantly moving in the fronto-parallel plane to give the visual impression of floating in the virtual environment. Subjects were explicitly instructed to take into account their prone position when making the passability judgment, which implicitly encouraged a behavioral context of floating horizontally and head-first through the doorway, rather than a context of upright, bipedal locomotion. Despite these manipulations to reduce potential effects of priming, we observed a robust effect of VEH on passability judgments and we found no difference between the moving viewpoint and a static presentation of the visual scene. This strongly suggests that a VEH strategy is universally applied regardless of the physical context. While we cannot exclude the possibility that weightlessness *plus* a floating visual context, or that more prolonged exposure to weightlessness could lead to a reduction in the reliance on VEH (the subject of ongoing experiments in lower Earth orbit), the bulk of existing evidence indicates that VEH influences on the perception of affordances is robust, being applied by human observers in a wide range of both common and unusual physical contexts. Preliminary results show that VEH effect persists during 6 month exposure to weightlessness [66]. From the experiments reported here, one can clearly conclude that physically standing on a support surface is not a necessary condition for the use of VEH as a cue to aperture size and passability.

### Practical Implications

Our study is interesting from a practical point of view in that visual sensorial substitution could be used to limit spatial disorientations for travelers during space flight [67] and perhaps in other disorienting conditions (e.g. scuba diving in enclosed spaces). Enhancing visual cues to orientation to provide a ground-like visual context could be a beneficial addition to the ergonomic design of spacecraft. For instance, it would be particularly valuable to consider the addition of visual directions such as static walls, floor, ceiling, and other fixed aspects of the immediate environment in module shuttles to restore a visual polarity. Furthermore, displaying visual landmarks such as the sky and ground, as well as the presence of an artificial horizon could also be considered in in-helmets visual display for extra-vehicular activities, since the observer would perceive the artificial horizon as the projection of his own eye-height [61].

## Conclusion

Taken together, our results demonstrate that visual eye-height is a robust cue which continues to be used for the perception of affordances during short exposure to weightlessness where the feet and the aperture do no longer share the same stable support surface. Moreover, our results also showed that the critical aperture is set as a fixed percentage of the subjective visual eye-height whatever the gravity or floor conditions. This suggest that in our experiment, the judgment of passability was not based solely on purely visual cues (e.g. the VEH), but could rely on more complex information (e.g. sVEH) integrating subjective metrics of the body.

## Supporting Information

S1 File Raw Data. Data used for statistics and figures.
(XLS)
